# An assessment of violent imagery in advertisements on city buses in Manhattan, New York City

**DOI:** 10.34172/hpp.2020.26

**Published:** 2020-03-30

**Authors:** Corey H. Basch, Jan Mohlman, Charles E. Basch

**Affiliations:** ^1^Department of Public Health, William Paterson University, Wayne, NJ 07470,USA; ^2^Department of Psychology, William Paterson University, Wayne, NJ 07470, USA; ^3^Richard March Hoe Professor of Health and Education, Department of Health and Behavior Studies, Teachers College, Columbia University, New York, NY 10027, USA

**Keywords:** Advertising, Violence, New York City, Aggression, Mass media

## Abstract

**Background:** Violence or violent imagery, defined as any image that conveys an imminent physical or existential threat to person(s), property, or society, with or without weaponry, is often featured in advertising. However, the effects of exposure (sporadic or chronic) to such imagery are not fully understood. The objective of this study was to describe the prevalence and types of violence portrayed in advertising on public buses in New York City (NYC).

**Methods:** In this cross-sectional study, from April to July 2019, researchers catalogued and coded the print advertising images present on the passenger entry side of all Metropolitan Transit Authority (MTA) bus lines in Manhattan to determine whether images of violence or violent acts were present. Unlike images of alcohol and tobacco products (banned from MTA property in 2017 and 1992, respectively), there are no similar restrictions on violence or violent imagery.

**Results:** A total of 23 out of 136 (17%) observed advertisements included images of violence and/or actual or imminent violent acts. One hundred percent of images involving violence were embedded in advertisements for mass media/entertainment purposes often featuring well known and favorably regarded actors and entertainment personalities or companies.

**Conclusion:** People of all ages and backgrounds are passively exposed to bus advertisements in a variety of settings. This study contributes to the literature regarding the extent to which the public is passively exposed to violent advertising. Additional study is required to further understand the link between violent imagery and attitudes toward/tolerance of violence.

## Introduction

Violence is a devastating public health problem with consequences for individuals, families and communities. Interpersonal violence is a widespread issue in the United States. For example, in 2017, the death rate from guns in the United States was 4.43 per 100 000 people, notably higher as compared to other countries.^1^ In New York City (NYC), 521 people were shot as a result of 441 incidents reported from January-July of 2019, an increase from prior years.^2^ Research suggests that exposure to violence, especially in earlier stages of life, may influence violent behavior,^3^ however studies of violence portrayed in advertisements on buses in NYC are lacking. Given that average weekday ridership is approximately two million people,^4^ the purpose of this study was to describe the prevalence and types of violence portrayed in advertising on public buses in NYC.

One argument in favor of efforts to control the prominence of violence in public settings can be found in the literature of desensitization, a process whereby emotional responses (e.g., anxiety, disgust) gradually or abruptly diminish or change across repeated exposures to violent content. It has long been recognized that desensitization has at least two possible emotional outcomes: reduced negative responses to violence (and sometimes enhanced enjoyment of violence) and reduced empathy for victims.^5^ These effects may last over several days, or are perhaps more durable under certain circumstances. The temporal aspects of desensitization are not well understood, however there are assumed to be aggregated effects of exposure across brief periods of time as well as over an individual’s lifetime.^5^ Furthermore, Jones and colleagues^6^ argue that because ads must capture attention, images are designed to be compelling, affecting the emotions, thoughts, and behaviors of viewers.^7^ In other words, there may be negative psychological effects of even the briefest exposures to violent images on buses.

There is a paucity of research regarding the extent to which violent advertisements appear on city buses. Therefore, the objective of this study was to describe the prevalence and types of violence portrayed in advertising on public buses in NYC. This will allow an estimate of the rate of passive societal exposure, information that is needed before estimates of the effects of such exposure can be estimated. If it discovered that violent bus ads are prevalent or memorable enough to affect individuals, then removal or restriction of violent images on buses could be a relatively simple and cost-effective method of reducing exposure to and thus, minimizing harmful effects of such violent images.

## Materials and Methods

Researchers adapted a cross-sectional approach that was used in their prior studies of advertising imagery on NYC subways.^8^ This study was ancillary to a study exploring alcohol imagery on buses, and is described in greater detail elsewhere.^9^ For each of the Metropolitan Transit Authority’s (MTA) 42 Manhattan bus lines, researchers observed and documented the advertising on the passenger side of the bus (as documenting traffic-side advertising posed a safety risk. In all, researchers observed 168 buses. Some buses (n = 32) did not have any advertising; these were excluded, leaving a sample of 136 buses and advertisements.

Because buses travel significant distances along their routes, researchers determined that just one observation point per route would be sufficient. At each observation point, a researcher photographed the advertisement on the side of the given bus and stored the data for coding and analysis. In addition, researchers recorded the distinctive identification number for each bus observed to ensure that each observation was of a unique bus. Buses were observed and data collected at various times of day, days of the week, and locations across Manhattan from April to July 2019.

Data was collected and coded using a spreadsheet instrument that was designed to capture relevant content characteristics of each advertisement. This instrument allowed the researchers to code and capture the object of each advertisement, to document whether images of violence or violent acts were present, and if so, what type of imagery was involved. When present, violent imagery was coded into one or more (where applicable) of five categories: Existential Threat, Violent Words, Weapon, Intent to Strike, or Destruction. These categories were based on our prior research on advertising present in publicly-owned/controlled spaces.^4^ Existential threat was defined as an image, idea, action, or event that instills or invokes feelings of personal or societal peril, either immediate or at some undetermined future time. These feelings could be strong or weak, and the perceived threat real or imagined; existential threats are defined by a personal feeling, not by an outside objective standard. Violent wording were words that communicated - either directly or indirectly - actions or ideas relating to harm to persons or property (e.g. hitman). Presence of a weapon indicated an object that could be used to inflict pain, injury, harm, or death on a person or object (guns, knives, swords, etc). Intent to strike was coded when a person was holding an object in such a way that the next action would be using the weapon to strike, harm, or otherwise injure a person or object. Destruction was defined as the act of conducting damage to property. The gender of the people in the advertisements were also coded. Multiple categories could be coded for each advertisement.

The researcher (CHB) reviewed all photographs to complete the initial coding; a second individual, researcher reviewed and coded a subset of 13 randomly selected advertisements to determine inter-rater reliability. There was 94% agreement. Descriptive statistics, including frequencies and percentages of the various categories of violent images were calculated using Microsoft Excel.

## Results

A total of 23 out of 136 (17%) of advertisements, including duplicates, with violence were observed in this study (95% CI: 10.6%, 23.2%). The overwhelming number of advertisements with violent images were for television shows (n = 18 of 23, 78%) with the remaining 22% (n = 5) for movie advertisements. The most common type of violent content displayed on city buses was presence of a weapon, followed by existential threat and intent to strike (Table 1). The majority of violent advertisements in this study featured men, although in many cases, the gender of the person appearing in the advertisement was not discernible.

## Conclusion

We found that violent images on buses are prominent in NYC, appearing on 1 out of every 6 buses. This rate of exposure to violent ads may be sufficient to affect attitudes of those who view them. Moreover, the majority of ads featured a weapon and/or an allusion to existential threat, defined as feelings of personal or societal peril, either immediate or at some undetermined future time. The impact of the current ads remains unclear, however certain factors facilitate the process of desensitization to violent images, such as explicitly contextualizing the images in a story,^10^ as was seen in all of violent bus ads studied herein.

It can be argued that the effects of repeated exposure to violent images in advertisements are cumulative, thus contributing to a longer-term process of desensitization, This can take place outside of conscious awareness, in everyday, seemingly mundane settings. For example, the crosstown bus route along 34^th^ Street in midtown Manhattan, NYC is populated by at least 100 buses per hour. Considering that 17% of bus ads display violent content, it can be deduced that individuals will be exposed to an ad featuring violence once every 3 to 4 minutes on 34^th^ Street. While certainly not as intensive as the rate of exposure to violent images one might experience in a laboratory, this overall amount of exposure would be equivalent to laboratory studies that used repeated short film clips (30 seconds to 2 minutes) to test desensitization in college students.^5^

Clearly more research is needed to determine the ultimate effects of violent bus advertisements, and whether it is attitudes about violence *per se* , or toward the subject of the ad (e.g., a featured television show or movie) that are most affected. There is also the matter of the impact of violent ads on the larger environment. Who pays the price of violent images found in the NYC transit system? It may be those with the most exposure to buses: their drivers. Between January and July of 2018, 962 incidents of aggression were reported by bus drivers.^11^ Partly in response to rising numbers of assaults on bus drivers, in June of 2019, the MTA, in conjunction with the Governor of New York announced that they would add 500 extra uniformed officers to the NYC Transit system. This was “part of a comprehensive action plan to improve safety across New York City’s mass transit system, address the rising number of assaults on transit workers and combat the growing problem of fare evasion.”^12^ It may be that advertising revenues are ironically undercut by the need to implement violence prevention for MTA workers.

This study had a number of limitations. We viewed buses in different neighborhoods at different times of day, most typically during business hours. Thus, we did not sample the range of ads occurring over consecutive 24 hour periods. Certain ads might be more likely to be on buses that arrive at locations at specific times, and there might be systematic differences in which ads appear on which buses at specific times of day. We sampled only on the passenger sides of buses, and passers-by might be more exposed to ads on the opposite side. Whether or not individuals are vulnerable to the psychological effects of incidental exposure to violent images is a complex issue. There are controlled intentional processes that can counteract such effects (e.g., an individual viewing an ad for an expensive watch could decrease desire for the watch by thinking of its prohibitive cost). Evidence suggests that controlled processes moderate brief exposures to ads even in the case of preconscious exposure. Therefore, it may be the case that only those who have pre-existing violent motives experience psychological effects of violent ads.^13^

Additional research is urgently needed to determine the type and severity of these effects, whether or not violent ad exposure should be considered a public health issue, and if regulating or banning violent ad content stands to benefit individuals and communities.

## Ethical approval

The Institutional Review Boards (IRB) at Teachers College, Columbia University determined that this study was exempt (Protocol # 19-328), while the IRB at William Paterson University does not review studies that do not involve human subjects.

## Competing interests

The authors of this study do not report any conflicts of interest.

## Funding

None declared.

## Authors’ contributions

CHB and CEB conceptualized the study. CHB collected the data. JM and CHB analyzed the data. All authors contributed to writing and revising the manuscript.

## Acknowledgments

The authors would like to acknowledge Kristina Berger for her contributions to this work.

**Table 1:** Violent content portrayed in 23 advertisements observed on city buses in Manhattan, New York City, 2019

**Content**	**N**	**% of 23 Advertisements**
Existential threat	11	48
Violent words	7	30
Weapon	12	52
Intent to strike	8	35
Destruction	8	35
